# Psychometric properties of outcome measurement instruments for ANCA-associated vasculitis: a systematic literature review

**DOI:** 10.1093/rheumatology/keac175

**Published:** 2022-03-16

**Authors:** Alvise Berti, Gonçalo Boleto, Peter A Merkel, Gunnar Tómasson, Sara Monti, Kaitlin A Quinn, Leslie C Hassett, Loreto Carmona, Sofia Ramiro

**Affiliations:** Santa Chiara Regional Hospital, APSS Trento, and Department of Cellular, Computational and Integrative Biology (CIBIO), University of Trento, Rheumatology, Trento, Italy; Rheumatology, Université de Paris, Hôpital Cochin, Paris, France; Division of Rheumatology, Department of Medicine, Division of Epidemiology, Department of Biostatistics, Epidemiology, and Informatics, Rheumatology Division, University of Pennsylvania, Philadelphia, PA, USA; Department of Rheumatology, and Centre for Rheumatology Research, University Hospital, Reykjavik, Iceland; Rheumatology Department, Fondazione IRCCS Policlinico San Matteo, University of Pavia, Italy; Systemic Autoimmunity Branch, National Institutes of Health, NIAMS, Bethesda, MD; Mayo Clinic Libraries, Mayo Clinic, Rochester, MN, USA; Rheumatology, Instituto de Salud Musculoesquelética (InMusc), Madrid, Spain; Rheumatology, Leiden University Medical Center (LUMC), Leiden; Rheumatology, Zuyderland Medical Center, Heerlen, The Netherlands

**Keywords:** ANCA-associated vasculitis, granulomatosis with polyangiitis, outcome measures, psychometric properties, OMERACT, disease activity

## Abstract

**Objectives:**

To systematically review the psychometric properties of outcome measurement instruments used in ANCA-associated vasculitis (AAV).

**Methods:**

Medline, EMBASE, Cochrane, Scopus and Web of Science were searched from inception to 14 July 2020 for validation studies of instruments used in AAV. Following the COnsensus-based Standards for the selection of health status Measurement INstruments (COSMIN) and OMERACT frameworks, different psychometric properties (validity, reliability, responsiveness and feasibility) were summarized. Risk of bias was assessed according to the COSMIN checklist.

**Results:**

From 2505 articles identified, 32 met the predefined selection criteria, providing information on 22 instruments assessing disease activity (*n* = 7), damage (*n* = 2), activity and damage (*n* = 1), health-related quality of life (HRQoL; *n* = 9) and function (*n* = 3). Most of the instruments were tested in AAV as a group or in granulomatosis with polyangiitis only.

The BVAS, any version, the Vasculitis Damage Index (VDI) and the AAV-Patient-Reported Outcome (AAV-PRO) have been more extensively validated than the other instruments. BVAS for Wegener Granulomatosis (BVAS/WG) has been shown to be valid for measuring disease activity [correlation with Physician global assessment (r = 0.90)], reliability (inter-observer intraclass correlation coefficient = 0.97), responsiveness and feasibility. For damage, VDI was shown to be moderately valid (correlations with BVAS version 3 at 6 months r = 0.14, BVAS/WG at 1 year r = 0.40 and 5 years r = 0.20), and feasible. For HRQoL, AAV-PRO demonstrated validity (correlations of the six AAV-PRO domains with EQ-5D-5L: −0.78 to −0.55; discrimination between active disease and remission, *P* < 0.0001 for all comparisons). The overall performance of instruments assessing function was low-to-moderate.

**Conclusion:**

Among the 22 outcome measurement instruments used for AAV, BVAS (any version), VDI and AAV-PRO had the strongest psychometric properties.

Rheumatology key messages

- Twenty-two outcome measurement instruments had their psychometric properties assessed in patients with AAV.
- In the majority of cases, instruments were tested in ANCA-associated vasculitis (AAV) as a group or in granulomatosis with polyangiitis only.
- The instruments with strongest psychometric properties were the BVAS (all versions) for disease activity, the Vasculitis Damage Index for damage and AAV-Patient-Reported Outcome (PRO) for PRO/quality of life.

## Introduction

ANCA-associated vasculitis (AAV) encompasses three major systemic clinical conditions caused by inflammation of the small blood vessels: granulomatosis with polyangiitis (GPA), eosinophilic granulomatosis with polyangiitis (EGPA) and microscopic polyangiitis (MPA, which includes the renal-limited form) [1]. GPA and MPA are characterized by heterogeneous manifestations and a great deal of clinical overlap between the two diseases. However, GPA has a greater predilection for the upper and lower respiratory tracts (with characteristic destructive lesions in the nasal septum, lung nodules and cavities), and MPA more frequently involves glomerulonephritis [1]. Asthma, nasal polyps and peripheral hyper-eosinophilia are unique features of EGPA, which represents ∼10–20% of patients with AAV, and has been treated as a separate clinical entity from GPA and MPA in clinical trials [1]. AAV often has a major impact on patients’ lives through both acute illness and over the long-term, affecting several major organs and threatening life [2].

To measure disease severity and response to treatment, and enable comparability across studies in AAV, defining standardized outcome measures is of utmost importance. This need has been well recognized by the OMERACT Vasculitis Working Group: a core set of domains and associated outcome measures have been endorsed to be used for AAV clinical trials [3, 4], i.e. disease activity, damage assessment, patient-reported outcomes (PRO) and mortality. Since the publication of the OMERACT core set for AAV, a substantial amount of additional research has been conducted on the performance of outcome measure instruments in AAV that assess various domains. For every instrument assessing the domains identified by OMERACT for AAV, the characteristics of the single instrument, such as the extent to which an instrument measures what it asserts to measure (i.e. validity), or the instrument’s ability to produce stable and consistent results (i.e. reliability), are collectively called psychometric properties.

OMERACT uses a staged process to establish core sets by first establishing the key domains of illness, and then identifying validated instruments to assess the domains [4], which is the result of a consensus expert opinion that did not rely on a systematic literature review of the available instruments used in AAV [5]. Systematic reviews of clinical trials and observational studies help catalogue outcome measures used and domains targeted for the disease of interest, inform groups to work towards agreement on relevant domains of illness and summarize the psychometric properties of instruments measuring each domain. Recently, a systematic review on the use and reporting of outcome measures in randomized clinical trials of AAV showed that a large degree of heterogeneity exists among instruments used in endpoint definitions and timing of assessments [6]. Therefore, to make informed choices of instruments to use to measure each domain, it would be useful to know the instruments’ psychometric properties.

The EULAR Outcomes Measures Library (OML) is an international collaborative initiative that is an open-access repository of outcomes measures in rheumatology [7] that uses the COnsensus-based Standards for the selection of health status Measurement INstruments (COSMIN) checklist to appraise the instruments [8]. One approach to populate the OML is through conducting systematic reviews of existing instruments for any given disease or domain and appraise the instruments’ psychometric properties.

Based on the interest of the vasculitis community of patients, clinicians and investigators in appraising the existing psychometric properties of instruments used for AAV, a systematic review was designed in collaboration between the OMERACT Vasculitis Working Group and the EULAR OML. We have reviewed and summarized the current evidence on psychometric properties of outcome measurement instruments used in AAV, covering each core domain as defined by OMERACT.

## Methods

The protocol of the systematic review was registered in PROSPERO (CRD42020209656). The review adheres to COSMIN guidelines [8, 9] and used the Preferred Reporting Items for Systematic Reviews and Meta-Analyses (PRISMA) guidelines for reporting [10].

### Search strategy and eligibility criteria

A comprehensive search in Ovid MEDLINE^®^ and Epub Ahead of Print, In-Process & Other Non-Indexed Citations, and Daily, Ovid EMBASE, Ovid Cochrane Central Register of Controlled Trials, Web of Science, and Scopus was conducted from each database’s inception to 14 July 2020.

The PIM framework (Population, Instrument of interest, Measurement properties) was used (Supplementary Material S1, available at *Rheumatology* online) [10]. The population was composed by patients with AAV: GPA; MPA; and EGPA. Any instrument covering a disease domain from the OMERACT core set was included. Articles were eligible if covering psychometric properties. Articles encompassing various systemic vasculitis but not presenting data for AAV separately were excluded (Supplementary Material S2, available at *Rheumatology* online).

Different psychometric properties were assessed: (i) validity [face validity, construct (group discrimination, hypothesis-testing or divergent/convergent validity), content and criterion validity], (ii) reliability (internal consistency, inter- and intra-observer reliability), (iii) responsiveness and (iv) feasibility (further details in Supplementary Material S3, available at *Rheumatology* online).

The search strategy was designed and conducted by an experienced librarian (L.C.H.) with input from the study's principal investigators. No limits on publication language or dates were imposed. Two reviewers (A.B., G.B.) screened independently titles and abstracts followed by full-text review of selected articles. Data extraction from papers was also independently performed by two investigators (A.B., G.B.). In case of disagreement, senior reviewers (S.R. and L.C.) helped to reach consensus.

### Data extraction

Data concerning study and instrument description and validation were collected (further details in Supplementary Material S4, available at *Rheumatology* online).

### Risk of bias assessment

Risk of bias was assessed according to the COSMIN checklist, a checklist that can be used at the level of the individual studies and of the instruments [8]. The studies were evaluated rating each property, when present, from ‘inadequate’ to ‘very good’ (not available, inadequate, doubtful, adequate, very good). The final risk of bias for each study was evaluated as ‘low’, ‘moderate’ or ‘high’ based on the evaluation of all the properties.

## Results

### Search results and study features

From 2505 references identified in the search, 156 were reviewed with full-text and 32 met the predefined selection criteria and were included in the final analyses (supplementary Fig. S1, available at *Rheumatology* online). The characteristics of the included studies, including instruments used, the OMERACT domains assessed, and risk of bias are reported in Table 1. Five studies focussed on the development of instruments [11–15], 24 were validation studies [16–39] and 3 pursued both objectives [40–42]. All studies involved an adult (≥18 years old) population except for one that focussed on a paediatric population with AAV [20]. The baseline characteristics of the study populations varied across the different studies, in terms of AAV subsets assessed, distribution by age and sex, sample size and country (supplementary Table S1, available at *Rheumatology* online).

**Table 1:** Description of the 32 studies assessing psychometric properties in patients with AAV

First author	Year	Instrument	Instrument abbreviation	Domain assessed	Objective	Study design	Population	Risk of bias
Morishita K.	2012	BVAS version 3	BVAS.v3	Disease activity	Validation	Cross-sectional	GPA, MPA, EGPA, unclassified	Low
Stone J.	2001	BVAS for Wegener Granulomatosis	BVAS/WG	Disease activity	Development and validation	Longitudinal	GPA	Moderate
Mahr A.	2008	BVAS for Wegener Granulomatosis	BVAS/WG	Disease activity	Development and validation	Longitudinal	GPA	Low
Kim M.K.	2020	Multivariable index for AAV	MVIA	Disease activity	Development	Longitudinal	GPA, MPA, EGPA	High
de Groot K.	2001	Disease Extent Index	DEI	Disease activity	Validation	Longitudinal	GPA	Low
Garske U.	2012	ENT assessment score	ENTAS	Disease activity, damage	Validation	Longitudinal	GPA	Moderate
Decker L.	2017	ENT assessment score-2	ENTAS2	Disease activity, damage	Validation	Longitudinal	GPA	Moderate
Del Pero M.	2013	Ear, Nose, Throat/GPA DAS	ENT/GPA DAS	Disease activity	Development	Cross-sectional	GPA	Moderate
Whiting-O’Keefe Q.E.	1999	Vasculitis activity index	VAI	Disease activity	Development	Longitudinal	GPA, EGPA, MPA	Low
Suppiah R.	2011	BVAS version 3	BVAS.v3	Disease activity	Validation	Cross-sectional	GPA, MPA, EGPA	Low
Specks U.	2013	BVAS, Vasculitis damage index, SF-36	BVAS, VDI, SF-36	Disease activity, damage, QoL/PRO	Validation	Longitudinal	GPA, MPA	Low
Yumura W.	2014	BVAS	BVAS	Disease activity	Validation	Longitudinal	MPA	Low
Metzler C.	2007	BVAS, Disease Extent Index	BVAS, DEI	Disease activity	Validation	Longitudinal	GPA	Low
Monach P.	2013	BVAS	BVAS/WG	Disease activity	Validation	Longitudinal	GPA, MPA	Low
Seo P.	2005	Vasculitis damage index	VDI	Damage	Validation	Longitudinal	GPA	Low
Itabashi M.	2014	Vasculitis damage index	VDI	Damage	Validation	Longitudinal	MPA	High
Robson J.	2015	Vasculitis damage index	VDI	Damage	Validation	Longitudinal	GPA, MPA	Low
Suppiah R.	2011	Combined Damage Assessment Index	CDA	Damage	Validation	Cross-sectional	GPA, EGPA, MPA	Low
Robson JC.	2018	AAV-Patient-Reported Outcome	AAV-PRO	HRQoL/PRO	Development	Longitudinal	GPA, MPA, EGPA	Low
Robson JC.	2018	AAV-Patient-Reported Outcome	AAV-PRO	HRQoL/PRO	Development and validation	Longitudinal	GPA, MPA, EGPA	Low
Tomasson G.	2012	Study Short-Form 36	SF-36	HRQoL/PRO	Validation	Longitudinal	GPA	Moderate
Tomasson G.	2014	Patient global assessment	PtGA	HRQoL/PRO	Validation	Longitudinal	GPA	Low
Tomasson G.	2019	Patient-Reported Outcome Measurement Information System	PROMIS	HRQoL/PRO	Validation	Longitudinal	GPA, MPA, EGPA	Low
Faurschou M.	2010	Study Short-Form 36	SF-36	HRQoL/PRO	Validation	Cross-sectional	GPA	Low
McClean A.	2016	Multidimensional Fatigue Inventory-20	MFI-20	HRQoL/PRO	Validation	Cross-sectional	GPA, MPA	Low
Thorpe C.T.	2007	Vasculitis Self-Management Scale	VSMS	HRQoL/PRO	Development	Longitudinal	GPA, EGPA, MPA	Low
Schwartz M.N.	2020	Brief Illness Perception Questionnaire	BIPQ	HRQoL/PRO	Validation	Longitudinal	GPA, EGPA, MPA	Low
Grayson P.C.	2013	Illness Perception Questionnaire, Multidimensional Fatigue Inventory-20	IPQ-R, MFI-20	HRQoL/PRO	Validation	Cross-sectional	GPA, EGPA, MPA	Low
Annapureddy N.	2016	Routine assessment of patient index data 3	RAPID3	Function	Validation	Longitudinal	GPA, MPA, EGPA	High
Moog P.	2016	Composite Autonomic Symptom Score 31	COMPASS31	Function	Validation	Cross-sectional	GPA, MPA	Moderate
Koutantji M.	2003	Health Assessment Questionnaire	HAQ	Function	Validation	Cross-sectional	GPA, MPA, EGPA	Low
Padoan R.	2018	Overall Disability Sum Score	ODSS	Function	Validation	Cross-sectional	EGPA	High

HRQoL/PRO: health-related quality of life/patient-reported outcomes; GPA: granulomatosis with polyangiitis, MPA: microscopic polyangiitis, EGPA: eosinophilic granulomatosis with polyangiitis.

### Risk of bias

The following studies had potential high risk of bias according to the COSMIN checklist: validation of the Routine Assessment of Patient Index Data 3 (RAPID3) [16], the multivariable index for AAV (MVIA) [12], the Ear, Nose and Throat (ENT)/GPA Disease Activity Score (ENT/GPA DAS) [11] and the Overall Disability Sum Score (ODSS) [26] (Table 1 for further details).

### Overview of the instruments’ psychometric properties assessed

The studies identified provided information on 22 instruments, 7 assessing disease activity, 2 assessing disease damage, 1 assessing both disease activity and damage, 9 assessing patient-reported outcomes and 3 assessing function.

Disease activity was assessed with BVAS and its revisions, the most widely accepted numeric scores for the assessment of disease-specific activity for AAV [20, 41, 42]; by ENT/GPA DAS, proposed for the assessment of disease activity in patients with otorhinolaryngological manifestations of GPA [11]; by Disease Extent Index (DEI), a validated instrument to quantitatively assessed disease extent and activity in patients with AAV [17]; and by MVIA and Vasculitis Activity Index (VAI), the first designed to estimate activity at diagnosis (and to predict all-cause mortality) in patients with AAV [12] and the second to incorporate appropriately weighted clinical measurements reflecting disease activity in systemic necrotizing vasculitis [15].

Disease damage was assessed with vasculitis damage index (VDI), a validated and widely used method for measuring damage sustained from vasculitis or its treatment [21], and Combined Damage Assessment Index (CDA), an instrument stemmed from the VDI that includes additional items of damage but not captured by individual items on the VDI [32]. ENT assessment score (ENTAS) and its newer version ENTAS 2, were both developed for a structured, reliable ENT assessment in patient with GPA and to evaluate disease activity and disease activity and damage, respectively [18, 19].

Health-related quality of life (HRQoL) was assessed by AAV-specific instruments, i.e. AAV-Patient-Reported Outcome (AAV-PRO) [13, 40], and Vasculitis Self-Management Scale (VSMS) [14], a measure of illness self-management for adults living with AAV; and by non-specific instruments, i.e. Patient-Reported Outcome Measurement Information System (PROMIS), a 10-item collection of self-reported health completed by vasculitis patients in 40–55 s [38]; Study Short-Form 36 (SF-36) [27, 43], a set of generic, coherent and easily administered quality-of-life measures; Multidimensional Fatigue Inventory-20 (MFI-20) [28], a 20-item scale designed to evaluate five dimensions of fatigue, i.e. general fatigue, physical fatigue, reduced motivation, reduced activity and mental fatigue; Patient Global Assessment (PtGA) assessed as 100-mm visual analogue scales [44]; Brief Illness Perception Questionnaire (BIPQ) [29], a nine-item scale designed to rapidly assess the cognitive and emotional representations of illness; and the revised Illness Perception Questionnaire (IPQ-R) [30], a recently developed revised version of the IPQ measuring the five coherent components that together make up the patient’s perception of their illness; and RAPID3 [16], an index of patient-reported measures completed by patients and calculated by a health professional in 5 s.

Function was assessed with non-AAV-specific instruments: HAQ [25, 45], a self-reported measure of functional status (disability) used in many diseases; overall disability sum score (ODSS) [26], an instrument used for disability in immune mediated polyneuropathies; and Composite Autonomic Symptom Score 31 (COMPASS31) [24], a generic instrument to assess autonomic symptoms across multiple domains.

**Fig. 1:**
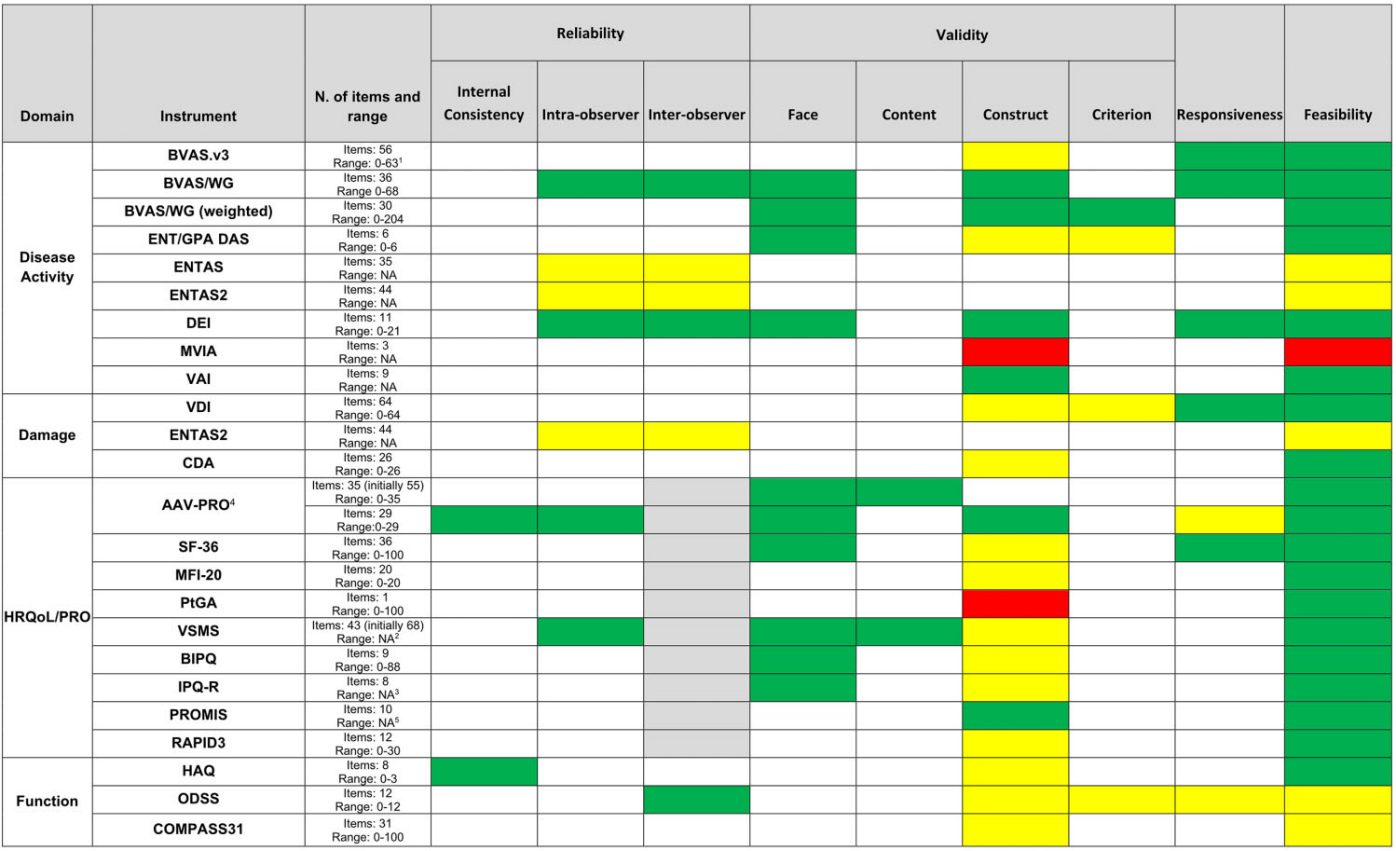
Overview of the psychometric properties of the 22 instruments used for AAV ^1^0–63 (new), 0–33 (persistent); ^2^1–5 each item (final score NA); ^3^0–22 domain ‘identity’, 1 to 5 Likert scale for the other 7 domains (final score NA); ^4^AAV-PRO: results are presented separately for the first version used for development (upper row) and for validation of the instrument (lower row); ^5^NA items banks of several domains. Color code: green means the performance of the instrument property tested it is considered adequate/good, yellow is considered moderate and red is considered low. Blank boxes means that the properties were not tested; grey boxes means that this psychometric property was not applicable to that specific instrument. N/A: not available; HRQoL/PRO: quality of life/patient-reported outcomes. Disease activity was assessed with BVAS version 3 (BVAS.v3) and BVAS for Wegener Granulomatosis (BVAS/WG), ENT/GPA DAS (ENT/GPA DAS), Disease Extent Index (DEI), multivariable index for AAV (MVIA) and Vasculitis Activity Index (VAI); disease damage was assessed with vasculitis damage index (VDI) and Combined Damage Assessment Index (CDA). ENT assessment score (ENTAS) and its newer version ENTAS 2 assessed both disease activity and damage. Health-related quality of life was assessed by AAV-specific instruments, i.e. AAV-Patient-Reported Outcome (AAV-PRO) and Vasculitis Self-Management Scale (VSMS), and non-specific instruments, i.e. Study Short-Form 36 (SF-36), Multidimensional Fatigue Inventory-20 (MFI-20), Patient Global Assessment (PtGA), Brief Illness Perception Questionnaire (BIPQ) and revised Illness Perception Questionnaire (IPQ-R), Patient-Reported Outcome Measurement Information System (PROMIS), Routine Assessment of Patient Index Data 3 (RAPID3). Function was assessed with non AAV-specific instrument, i.e. HAQ, Overall Disability Sum Score (ODSS) and Composite Autonomic Symptom Score 31 (COMPASS31). Colour version available online.

The psychometric properties of the 22 instruments are summarized in Fig. 1. There was a wide heterogeneity in the psychometric properties assessed for each instrument. A few psychometric properties have been considered in each study, with validity being the most frequently assessed aspect, in 82% of the instruments, but few properties other than construct validity were reported. Overall, the BVAS for disease activity, the VDI for damage, and the AAV-PRO for HRQoL/PRO, were the instruments with the best performance within the psychometric properties assessed (Fig. 1).

### Instruments tested in different AAV subsets

**Fig. 2.**
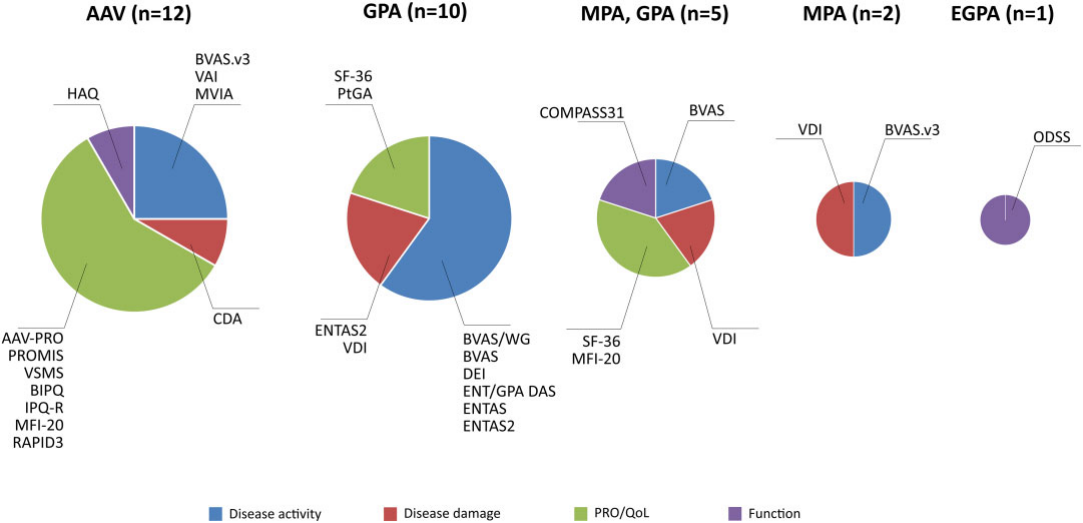
Instruments tested in the different subsets of AAV (all AAV, GPA, MPA, GPA and MPA, EGPA) by the OMERACT domain assessed (disease activity, damage, PRO/HRQoL and function) The sizes of the circles represent the total number of articles with psychometric properties assessed in AAV or its subsets (the number is reported int the brackets), the slice represent the percentage of articles assessing the specific instruments in that group of AAV or subset of AAV. Disease activity was assessed with Birmingham Vasculitis Activity Score (BVAS) version 3 (BVAS.v3) and BVAS for Wegener Granulomatosis (BVAS/WG), Ear nose and throat (ENT)/GPA disease activity score (ENT/GPA DAS), Disease Extent Index (DEI), multivariable index for AAV (MVIA) and Vasculitis Activity Index (VAI); disease damage was assessed with vasculitis damage index (VDI) and Combined Damage Assessment Index (CDA). ENT assessment score (ENTAS) and its newer version ENTAS 2 assessed both disease activity and damage. Health-related quality of life was assessed by AAV-specific instruments, i.e. AAV-Patient-Reported Outcome (AAV-PRO) and Vasculitis Self-Management Scale (VSMS), and non-specific instruments, i.e. Study Short-Form 36 (SF-36), Multidimensional Fatigue Inventory-20 (MFI-20), Patient Global Assessment (PtGA), Brief Illness Perception Questionnaire (BIPQ), and revised Illness Perception Questionnaire (IPQ-R), Patient-Reported Outcome Measurement Information System (PROMIS), Routine Assessment of Patient Index Data 3 (RAPID3). Function was assessed with non AAV-specific instrument, i.e. Health Assessment Questionnaire (HAQ), Overall Disability Sum Score (ODSS) and Composite Autonomic Symptom Score 31 (COMPASS31). GPA: granulomatosis for polyangiitis; MPA: microscopic polyangiitis; EGPA: eosinophilic granulomatosis with polyangiitis. Colour version available online.

Most of the instruments were tested in AAV as a group (i.e. including GPA, MPA and EGPA) or GPA only, followed by MPA and GPA, MPA only and EGPA only. Fig. 2 represents the instruments tested in the different AAV subsets by the OMERACT domain assessed. Among others, the BVAS version 3 (BVAS.v3) was validated in all AAV, while BVAS for Wegener Granulomatosis (BVAS/WG) in GPA and MPA, and the DEI in GPA only. Specific studies aiming to validate BVAS and VDI have not yet been performed in EGPA only. AAV-PRO was developed and validated in all forms of AAV, while the ODSS has been validated in EGPA only.

### Validity

Validity was analysed very differently across studies, making comparisons more difficult (Table 2). For disease activity, BVAS/WG demonstrated the most adequate construct validity, having the highest correlation with Physician Global Assessment (PhGA; r = 0.92 and r = 90 in the weighted version). The DEI and BVAS.v3 followed the BVAS/WG but did not have the same level of construct validity (for example, BVAS.v3 had lower correlation with PhGA, r = 0.38, compared with the BVAS/WG). The MVIA has weak construct validity (correlation with BVAS r = 0.37). The VAI for disease activity and the CDA for damage were shown to differ significantly across different type of vasculitis, but besides discriminant validity, no other psychometric measures have been tested in patients with AAV for these two instruments.

**Table 2:** Summary of the results on validity of the instruments used for AAV retrieved in the search

Domain	Instrument	Validity (face, content, construct discriminating, construct hypothesis-testing, criterion)
Disease activity	BVAS.v3	**Discriminating:** Patients with AAV *vs* other vasculitides (median, range): GPA (general): 1 (0, 36); GPA (non-renal): 0 (0, 39); EGPA: 0 (0, 14); MPA: 2 (0, 22); others: 0 (0, 15); MC: 5 (0, 26); HScP: 1 (0, 13); TAK: 0 (0, 4); Behçet: 6 (0, 18); SV: 2.5 (2, 3); PAN: 0.5 (0, 1)
		**Hypothesis-testing:** PhGA r = 0.379 (95% CI 0.233, 0.509, *P *< 0.0001); treatment decision r = 0.521 (95% CI 0.393, 0.629, *P *< 0.0001); ESR r = 0.403 (95% CI 0.253, 0.533, *P *< 0.0001)
	BVAS/WG	**Face:** yes
		**Hypothesis-testing:** PhGA, r = 0.92 (95% CI 0.89, 0.94), PhGA (only 86 active cases): r = 0.81 (0.73–0.87); ESR, r = 0.28, *P *= 0.0006; CRP, r = 0.22, *P *= 0.008
	BVAS/WG (weighted)	**Face:** yes
		**Hypothesis-testing:** PhGA r = 0.90, *P *< 0.0001
		**Criterion** (*vs* PhGA)**:** BVAS R^2^: 0.6732 (linear regression models, data-driven weighting)
	ENT/GPA DAS	**Face:** yes
		**Hypothesis-testing:** Bloody rhinorrhea rho 0.456 (*P *< 1.0^–6^); abnormal rhinoscopy rho 0.513 (*P *< 1.0^–6^); abnormal rhinoscopy (without crusting) rho 0.384 (*P *< 2.0^–6^); stridor as sign rho 0.450 (*P *< 1.0^–6^); abnormal laryngoscopy rho 0.454 (*P *< 1.0^–6^)
		**Criterion:** AUC ENT/DAS: sensitivity 97.8%, specificity 52.6%; compared with ENT/BVAS (gold standard): sensitivity 45.5%, specificity 89.6%
	DEI	**Face:** yes
		**Discriminating:** Between patients with AAV with active disease (T1) versus patients with partial or complete remission (T2): mean (s.d.)/median (range): 8.6 (2.7)/9 (2; 13) *vs* 2.2 (1.9)/2.2 (0; 7)
		**Hypothesis-testing:** T1 (active disease, *n* = 35): BVAS, rho = 0.90, *P *= 0.001; cANCA, rho = 0.46, *P *= 0.006; CRP, rho = 0.28, *P *> 0.05; IL-2R, rho = 0.07, *P *> 0.05; leucocyte count, rho = 0.38, *P *= 0.026; platelet count, rho = 0.53, *P *= 0.001
		**Hypothesis-testing:** T2 (remission, *n* = 35): BVAS, rho* *= NR, *P *> 0.05; cANCA, rho = 0.61, *P *= 0.001; CRP, rho = 0.47, *P *= 0.005; IL-2R, rho = 0.47, *P *= 0.0016; leukocyte, rho* *= –0.34, *P *> 0.05; platelet count, rho = 0.15, *P *> 0.05
	MVIA	**Discriminating:** Severe AAV (BVAS ≥16): AUROC curve 0.727 (95% CI 0.648, 0.805), optimal cut-off 1.35 (sensitivity 0.667, specificity 0.689)
		**Hypothesis-testing:** BVAS (at diagnosis) r = 0.370, *P* < 0.001
	VAI	**Discriminating:** Significant differences across the types of vasculitis (mean; min, max): relapsing polychondritis: 0.52 (0.15, 1.06); MPA: 0.65 (0.00, 3.41); other SNV: 0.83, (0.00, 2.96); EGPA: 0.78 (0.00, 1.69); GPA: 0.77 (0.00, 2.44); Behçet’s: 0.90 (0.00, 3.70); cryoglobulinemic vasculitis: 0.94 (0.00, 2.99)
Damage	VDI	**Hypothesis-testing:** BVAS/WG at baseline (with VDI at 1 year) r = 0.20, *P *= 0.015; SF-36 PCS r = –0.31 *P *< 0.0001, MCS r = –0.45 for limited disease and r = 0.059 for severe disease, *P *< 0.01; BVAS at diagnosis (with VDI score at 5 years) r = 0.4, *P *= 0.04. BVAS at baseline and VDI score at 6 months (r = 0.139, *P *= 0.001) and VDI score at long-term follow-up (r = 0.139, *P *= 0.016)
		**Discriminating:** Association with number of flares at 1 year: 0.47 ± 0.17 for VDI = 0, 0.72 ± 0.15 for VDI = 1, 1.27 ± 0.19 for VDI = 2, 0.65 ± 0.21 for VDI = 3, 1.48 ± 0.26 for VDI ≥ 4 (*P *= 0.012); association with number of serious adverse events 0.57 ± 0.35 for VDI = 0, 0.89 ± 0.30 for VDI = 1, 0.85 ± 0.38 for VDI = 2, 2.91 ± 0.43 for VDI = 3, 3.24 ± 0.52 for VDI ≥ 4 (*P *= 0.049); relapse group versus the non-relapse group (6.5 ± 2.3 *vs* 4.0 ± 1.0; *P *= 0.02) at 5 years
	CDA	**Discriminating:** Differences across the types of vasculitis (mean; min, max): GPA (renal): 4 (0, 26); GPA (non-renal): 4 (0, 26); MPA: 3 (0, 12); EGPA: 3 (0, 15); other: 2 (0, 16); HScP: 1 (0, 11); Mixed cryoglobulinemia: 4.5 (0, 10); Behçet’s: 5 (1, 8); TAK: 4 (0, 7); Isolated skin vasculitis: 0.5 (0, 9); PAN: 1 (0, 2); rheumatoid vasculitis: 2 (2, 2)
HRQoL/PRO	AAV-PRO	**Face:** yes
		**Content:** Smith’s Salience Index
		**Discriminating:** Between patients with AAV who are active versus in remission (self-identified) [mean (s.d.)]: OSS = 47.28 (22.55) *vs* 29.35 (21.86), *P *< 0.0001; SSS = 60.75 (25.37) *vs* 35.53 (25.70), *P *< 0.0001; TSE = 48.54 (22.12) *vs* 30.59 (20.09), *P *< 0.0001; SEI = 53.54 (24.17) *vs* 35.65 (24.69), *P *< 0.0001; CAF = 56.76 (24.39) *vs* 38.50 (25.52), *P *< 0.0001; PF = 44.08 (22.76) *vs* 27.56 (24.49), *P *< 0.0001
		**Hypothesis-testing:** EQ-5D-5L (tested on a subset): OSS r = −0.55, *P *< 0.001; SSS r = −0.67, *P *< 0.001; TSE r = −0.65, *P *< 0.001; SEI r = −0.73, *P *< 0.001; CAF r = −0.68, *P *< 0.001; PF r = −0.78, *P *< 0.001
		**Discriminating:** Patients with AAV versus age- and sex-matched controls: Physical function 72.5 (25.9) *vs* 88.0 (18.1), *P *< 0.001; Role physical 57.7 (45.3) *vs* 82.8 (32.2), *P *< 0.001; Bodily pain 72.4 (28.5) *vs* 79.6 (22.8), *P *= 0.09; General health 72.4 (28.5) *vs* 79.6 (22.8), *P *< 0.001; Vitality 61.9 (22.7) *vs* 72.8 (19.8), *P *< 0.001; Social functioning 80.9 (23.3) *vs* 93.0 (15.9), *P *< 0.001; Role emotional 73.0 (35.3) *vs* 87.2 (29.0), *P *< 0.001; Mental health 78.4 (17.9) *vs* 85.3 (14.6), *P *< 0.001; PCS score 44.9 (10.5) *vs* 52.0 (8.6), *P *< 0.001; MCS score 51.5 (9.3) *vs* 55.1(8.2), *P *< 0.001
		**Hypothesis-testing:** Total VDI (with SF-36 and subscales): no correlation; Pulmonary VDI damage (with physical functioning subscale): rho* *= −0.292, *P *= 0.02; Pulmonary VDI damage (with bodily pain subscale): rho* *= −0.298, *P *= 0.01; other organ-specific VDI scores: no correlations; BVAS/WG: 1-unit increase in BVAS/WG corresponds to: PCS −1.15 (95% CI −1.29, −1.02) in the WGET and –1.06 (95% CI −1.31, −0.82) in the VCRC-LS; MCS –0.93 (95% CI −1.07, −0.78) in the WGET and –0.89 (95% CI −1.20, −0.58) in the VCRC-LS; VDI: 1-unit increase in VDI (corrected for BVAS/WG) corresponds to: PCS –0.91 (95% CI –1.38, –0.44) in the WGET cohort, no association with PCS in VCRC-LS; no association of MCS in both WGET and VCRC-LS cohorts
	SF-36	**Face:** yes
	MFI-20	**Discriminating:** Patients with AAV *vs* healthy controls (median, interquartile range): 13 (8–16) *vs* 5.5 (4–8), *P *< 0.001
		**Discriminating:** Patients with AAV *vs* other vasculitides (median, range): Behçet’s: 17.0 (7–20); PCNSV: 16.0 (4–20); EGPA: 16.1 (4–20); GCA: 16.0 (4–20); GPA: 15.0 (4–20); HScP: 14.5 (4–20); MPA: 15.5 (4–20); PAN 16.0 (4–20); TAK: 16.0 (4–20).
		**Hypothesis-testing:** Anxiety score (HADS) r = 0.32, *P *< 0.001; Depression score (HADS) r = 0.57, *P *< 0.001; Global PSQI score r = 0.32, *P *< 0.001; Pain r = 0.27, *P *< 0.001
	PtGA	**Hypothesis-testing:** PhGA r = 0.30, *P *< 0.0001; BVAS/WG, r = 0.28, *P *< 0.0001; SF-36 items: PCS r = –0.38, MCS r = −0.30, both *P *< 0.0001
	VSMS	**Face:** yes
		**Content:** Principal component analysis followed by pairwise correlation matrix and then Cattell’s scree test and parallel analysis
		**Hypothesis-testing:** Correlation of the 8 subscales with ‘Marlowe-Crowne Social Desirability Scale’ assessing for social desirability bias: Medication adherence: r = 0.26, *P *< 0.01; Health services: r = 0.15, *P *< 0.05; Infection avoidance: r = 0.15, *P *> 0.05; Diet: r = 0.14, *P *> 0.05; Exercise: r = 0.05, *P *> 0.05; Symptom monitoring: r = 0.22, *P *< 0.05; Reporting symptoms and side effects: r = 0.19, *P *< 0.05; Adjusting activities: r = 0.17, *P *< 0.05. Correlation of the 8 subscales with ‘General Adherence Scale’ scores, assessing adherence: Medication adherence: r = 0.26, *P *< 0.01; Health services: r = 0.13, *P *> 0.05; Infection avoidance: r = 0.10, *P *> 0.05; Diet: r = 0.31 *P *< 0.01; Exercise: r = 0.39, *P *> 0.01; Symptom monitoring: r = 0.26, *P *< 0.01; Reporting symptoms and side effects: r = 0.20, *P *< 0.01; Adjusting activities: r = 0.05, *P *> 0.05
	BIPQ	**Face:** yes
		**Discriminating:** Differences across the four types of vasculitis: AAV: 31.13 (s.d. = 12.16); GCA: 35.16 (s.d. = 13.93); TAK: 38.4 (s.d. = 14.47); RP: 52.11 (s.d. = 10.36) (*P *< 0.0001)
	IPQ-R	**Face:** yes
		**Discriminating:** Differences of each item across the types of vasculitis:
		*Identity*: Behçet’s 13.5 (5.3); PCNSV 11.7 (4.5); EGPA 10.5 (4.9); GCA 8.7 (4.7); GPA 10.9 (5.2); HScP 5.7 (3.0); MPA 10.9 (5.3); PAN 10.9 (4.0); TAK 9.2 (5.0)
		*Timeline (acute–chronic)*: Behçet’s 4.2 (0.6); PCNSV 4.3 (0.9); EGPA 4.2 (0.8); GCA 3.7 (0.7); GPA 4.1 (0.8); HScP 3.3 (1.0); MPA 4.0 (0.9); PAN 4.2 (0.7); TAK 3.9 (0.9)
		*Timeline (cyclical)*: Behçet’s 4.0 (0.6); PCNSV 3.4 (1.1); EGPA 3.1 (0.9); GCA 3.0 (0.9); GPA 3.1 (1.0); HScP 3.0 (1.0); MPA 3.1 (1.1); PAN 3.6 (0.9); TAK 3.2 (0.8)
		*Consequences*: Behçet’s 4.0 (0.7); PCNSV 4.6 (0.4); EGPA 3.9 (0.8); GCA 3.7 (0.8); GPA 3.8 (0.9); HScP 3.1 (0.8); MPA 3.7 (0.9); PAN 4.0 (0.8); TAK 3.9 (0.8)
		*Personal control:* Behçet’s 3.1 (0.7); PCNSV 2.8 (0.8); EGPA 3.3 (0.9); GCA 3.0 (0.8); GPA 3.3 (0.8); HScP 3.2 (0.8); MPA 3.4 (0.7); PAN 3.2 (0.8); TAK 3.4 (0.7)
		*Treatment control*: Behçet’s 3.0 (0.7); PCNSV 3.0 (1.0); EGPA 3.2 (0.7); GCA 3.5 (0.7); GPA 3.4 (0.7); HScP 3.3 (0.5); MPA 3.4 (0.7); PAN 3.2 (0.8); TAK 3.4 (0.7)
		*Emotional representations*: Behçet’s 3.4 (0.8)/PCNSV 3.7 (0.9)/EGPA 3.2 (1.0); GCA 3.2 (1.0); GPA 3.0 (0.9); HScP 2.8 (0.9); MPA 3.2 (1.0); PAN 3.5 (0.9); TAK 3.0 (0.9)
		*Illness coherence*: Behçet’s 3.2 (1.0); PCNSV 3.0 (1.1); EGPA 3.4 (1.0); GCA 3.3 (1.2); GPA 3.5 (0.9); HScP 3.1 (1.1); MPA 3.3 (1.0); PAN 3.2 (1.0); TAK 3.4 (0.9)
	PROMIS	**Discriminating:** Scores on measures of PROMIS measures were overall reduced compared to US population norms: PROMIS measures worse (i.e., higher) than the population norms for fatigue, pain interference and sleep-related impairment, and worse (i.e., lower) for physical function and social isolation. Measures for cognitive abilities, social participation, anger and anxiety: close to the population norms. No substantial differences in PROMIS scores across different type of vasculitis
	RAPID3	**Discriminating:** Between patients with active (BVAS >0) and inactive (BVAS = 0) disease, in 4 different follow-up visits: visit 1 (V1): 7.0 *vs* 3.0, *P *= 0.115; V2: 8.8 *vs* 1.0, *P *= 0.011; V3: 6.1 *vs* 2.0, *P *= 0.032; V4: 11.7 *vs* 2.0, *P *= 0.128
		**Hypothesis-testing:** BVAS r = 0.42, *P *= 0.01
Function	HAQ	**Discriminating:** Between AAV and age- and sex-matched patients with RA: higher HAQ and HAQ square root in RA patients (values NA), *P *< 0.001 and *P *< 0.01, respectively
		**Hypothesis-testing:** SF-36: PCS r = −0.80 (*P *< 0.001) and MCS r = −0.37 (*P *< 0.01); VDI r = 0.15 (*P *> 0.05)
	ODSS	**Hypothesis-testing:** VDI ‘concordant r* *= NR’, *P *= 0.0063
		**Discriminating:** Neurological relapse ODSS > 3 53.8% (*n* = 7) *vs* ≤3 0%, *P *= 0.027
	COMPASS31	**Discriminating:** Patients with AAV versus healthy controls, median of the total COMPASS31 scores: 10.4 versus 3.0, *P *= 0.005
		**Hypothesis-testing:** BVAS ‘no correlation, r* *= NR’, *P *> 0.05

AAV: ANCA-associated vasculitis; AUC: area under the curve; AUROC curve: area under the receiver operating characteristic curve; GPA: granulomatosis with polyangiitis; MPA: microscopic polyangiitis; EGPA: eosinophilic granulomatosis with polyangiitis; MC: mixed essential cryoglobulinemia; SV:  (Leucocytoclastic) skin vasculitis; PAN: polyarteritis nodosa; TAK: Takayasu arteritis; PCNSV: primary CNS vasculitis; WGET: Wegener’s Granulomatosis Etanercept Trial; VCRC-LS: Vasculitis Clinical Research Consortium Longitudinal Study; NR: non reported; NA: not available; HRQoL/PRO: health-related quality of life/patient-reported outcomes. Instruments: BVAS.v3: BVAS version 3; BVAS/WG: BVAS for Wegener Granulomatosis; ENT/GPA DAS: Ear, Nose, Throat/GPA DAS; DEI: Disease Extent Index; MVIA: multivariable index for AAV and VAI: Vasculitis Activity Index; VDI: Vasculitis Damage Index; CDA: Combined Damage Assessment Index; ENTAS: ENT Assessment Score and ENTAS 2, its newer version; AAV-PRO: AAV-Patient-Reported Outcome; VSMS: Vasculitis Self-Management Scale; SF-36: Study Short-Form 36 (PCS: Physical Component Summary; MCS: Mental Component Summary); MFI-20: Multidimensional Fatigue Inventory-20; PhGA: Physician Global Assessment; PtGA: Patient Global Assessment; BIPQ: Brief Illness Perception Questionnaire; IPQ-R: revised Illness Perception Questionnaire; RAPID3: Routine assessment of patient index data 3; ODSS: Overall Disability Sum Score; COMPASS31: Composite Autonomic Symptom Score 31; HADS: Hospital Anxiety and Depression Scale; EQ-5D-5L: EuroQol-5D-5L; OSS: Organ Symptoms Severity; SSS: Systemic Symptoms Severity; TSE: Treatment Side-Effects; SEI: Social and Emotional Impact; CAF: Concerns About the Future; PF: Physical Functionl PROMIS: Patient Reported Outcome Measurement Information System.

For damage, the VDI was shown to have some construct validity, but its adequacy was low (correlations with BVAS.v3 at 6 months r = 0.14, BVAS/WG at 1 year r = 0.40 and 5 years r = 0.20). AAV-PRO is a PRO specifically developed to assess HRQoL in patients with AAV. AAV-PRO had the best performance for validity (construct validity: correlations of the six AAV-PRO domains with EQ-5D-5L: −0.78 to −0.55; discrimination validity: discrimination between active disease *vs* remission, *P* < 0.0001 for all comparisons; and high face validity and content validity: Smith’s Salience Index was used to identify the most salient items). The validity was overall moderate for the other AAV-specific instruments (VSMS, since it was possible to extrapolate data specific for AAV only for discriminant validity and not for construct validity, one of the aims of the study) and several instruments not specific for AAV assessing HRQoL/PRO (PROMIS, SF-36, MFI-20, PtGA, BIPQ, IPQ-R and RAPID3) and function (HAQ, ODSS and COMPASS31).

### Reliability

For disease activity, BVAS/WG was shown to have the highest intraclass correlation coefficient (ICC) (ICC = 0.97), followed by DEI (ICC = 0.96), while for function ODSS was shown to have the highest ICC (ICC = 0.96). ENTAS and ENTAS 2 each have moderate inter- and intra-observer reliability, while the instrument domains of AAV-PRO and VSMS have intra-observer reliability ICCs ranging between 0.89 and 0.96, and between 0.51 and 0.76, respectively (Table 3). Reliability has not been assessed for the VDI. Internal consistency was demonstrated for both the AAV-PRO and the HAQ (Cronbach’s alphas 0.77–0.96 and 0.91–0.93, respectively).

**Table 3:** Summary of the results on reliability of the instruments used for AAV retrieved in the search

Domain	Instrument	Reliability (internal consistency, intra-observer, inter-observer)
Disease activity	BVAS/WG	**Intra-observer:** ICC = 0.62
		**Inter-observer:** ICC = 0.97
	ENTAS	**Intra-observer:**
		**Cohen’s K (dichotomized):** K = 0.58 (inexperienced physicians) and 0.72 (experienced physicians);
		**Grading:** none, mild, moderate, high: K = 0.67 (inexperienced physicians) and 0.80 (experienced physicians)
		**Inter-observer:**
		**Fleiss’s K:** K = 0.62 for T1 and K = 0.59 for T2 (inexperienced physicians); K = 0.50 at T1 and K = 0,58 at T2 (experienced physicians)
		**Grading:** none, mild, moderate, high: ICC = 0.69 for T1 and ICC= 0.59 for T2 (inexperienced physicians); ICC= 0.77 for T1 and ICC = 0.75 for T2 (experienced physicians)
	ENTAS 2	**Intra-observer:** K = 0.56
		**Inter-observer:** K = 0.43 at T1 and K = 0.48 at T2
	DEI	**Intra-observer:** r = 0.94, *P *= 0.0001 (subset of 21 patients)
		**Inter-observer:** r = 0.96, *P *= 0.0001 (subset of 21 patients)
Damage	ENTAS 2	**Intra-observer:** K = 0.74
		**Inter-observer:** K = 0.79 at T1 and K = 0.64 at T2
HRQoL/PRO	AAV-PRO	**Internal consistency:** Cronbach’s alpha 0.77–0.96
		**Intra-observer:** Three-to-five days later, only US sample: OSS ICC = 0.89 (95% CI 0.84–0.93); SSS ICC = 0.91 (0.86–0.94); TSS = 0.95 (0.93–0.97); SEI = 0.96 (0.94–0.97); CAF = 0.95 (0.92–0.97); PF = 0.96 (0.94–0.97).
	VSMS	**Intra-observer:** Medication adherence: ICC 0.61; Health services adherence: ICC 0.54; Infection avoidance adherence: ICC 0.69; Diet adherence: ICC 0.60
		Exercise adherence: ICC 0.75; Symptom monitoring adherence: ICC 0.63; Reporting symptoms and side effects: ICC 0.76; Adjusting activities: ICC 0.76
Function	HAQ	**Internal consistency:** Cronbach’s alpha 0.91–0.93
	ODSS	**Inter-observer:** K = 0.96

ICC: intraclass correlation coefficient; HRQoL/PRO: health-related quality of life/patient-reported outcomes. Instruments: BVAS.v3: BVAS version 3 and BVAS/WG: BVAS for Wegener Granulomatosis; DEI: Disease Extent Index; VDI: vasculitis damage index; ENTAS: ENT Assessment Score and its newer version ENTAS 2; AAV-PRO: AAV-Patient-Reported Outcome; VSMS: Vasculitis Self-Management Scale; ODSS: Overall Disability Sum Score; OSS: Organ Symptoms Severity.

### Responsiveness

BVAS.v3, BVAS/WG, VDI and SF-36 have been shown to be sensitive to change in randomized controlled trials of AAV (Table 4). DEI has a mean standardized response of 2.37 s.d. units, while in AAV-PRO responsiveness was moderate (effects size ranging from 0.0 to 0.09 for ‘no change’ *vs* from 0.21 to 0.28 for ‘much better’), likely limited by the short time-interval of 3 months in patients that were in remission in 70% of cases (and therefore were not expected to change in clinical state, as in the context of a clinical trial). ODSS has been shown to change moderately during follow-up from baseline [baseline to 6 months (4.2 ± 2.4–2.9 ± 1.5, *P* = 0.0001)].

**Table 4:** Summary of the results on responsiveness of the instruments used for AAV retrieved in the search

Domain	Instrument	Responsiveness
Disease activity	BVAS.v3	**Intrinsic:** Baseline BVAS (inactive disease): median 0 (range 0, 1) to 7.5 (5, 9) in LEF group and from 0 (0, 4) to 10.5 (1, 24) in MTX group during relapse (active disease)
		Baseline BVAS (active disease): 12.2 ± 9.1–0 ± 0 after induction therapy
	BVAS/WG	**Intrinsic:** BVAS/WG after induction (remission): median (interquartile range) 0 (0, 0) by definition, during relapse (active disease): 2.5 (2.0, 4.5) in RTX group and 4.0 (2.0, 4.5) in CYC group
	DEI	**Intrinsic:** Mean standardized response: 2.37 units (considerable change)
		Baseline DEI (inactive disease): median 0 (range 0, 4) to 4 (2, 14) in LEF group and to 3.5 (2, 7) in MTX group during relapse (active disease)
Damage	VDI	**Intrinsic:** VDI mean change from baseline to month 18: 1.3 ± 1.59 in RTX group, 1.3 ± 1.43 in CYC group
HRQoL/PRO	AAV-PRO	**Minimal Detectable Change (MDC90) Raw score:** Organ-specific symptoms 3.64, systemic symptoms 3.08, treatment side effects 2.31, social and emotional impact 2.91, concerns about the future 2.79, physical function 1.94
		**Effect size (responses):** Much better (0.21, 0.28), slightly better (0.01, 0.19), no change (0.00, 0.09) slightly worse (–0.19, 0.04), much worse (–0.45, 0.06)
	SF-36	**Intrinsic:** Mean (s.d.) change of physical component SF-36 at month 18 from baseline: 9.3 (10.96) in RTX group, 9.3 (11.45) in CYC group
		Mean (s.d.) change of mental component SF-36 at month 18 from baseline: 11.6 (12.23) in RTX group, 9.0 (11.18) in CYC group
Function	ODSS	**Intrinsic:** From baseline to 6 months (4.2 ± 2.4–2.9 ± 1.5, *P *= 0.0001), from baseline to 12 months (2.6 ± 1.5) and to last follow-up (2.2 ± 1.2)

HRQoL/PRO: quality of life/patient-reported outcomes; RTX: rituximab, LEF: leflunomide. Instruments: BVAS.v3: BVAS version 3 and BVAS/WG: BVAS for Wegener Granulomatosis; DEI: Disease Extent Index; VDI: vasculitis damage index; AAV-PRO: AAV-Patient-Reported Outcome; Vasculitis; SF-36: Study Short-Form 36; ODSS: Overall Disability Sum Score.

### Feasibility

The majority of instruments were shown to be feasible, except for two AAV-specific instruments, the ENTAS and the ENTAS 2, due to complexity of the instrument, time needed, necessity of training and raters limited to ENT specialists; and two non-AAV specific instruments, the ODSS and the COMPASS31, due to the complexity of the instruments and necessity for training.

## Discussion

This is the first systematic review summarizing the psychometric properties of outcome measurement instruments developed or validated for AAV. Twenty-two instruments covering the OMERACT domains of disease activity, damage, QoL/PRO and function have had their psychometric properties assessed. The domains identified in this systematic review are endorsed by OMERACT as the core set outcomes for randomized controlled trials of AAV [3, 5, 38, 46]. The majority of instruments were developed or validated in AAV as a group or in GPA only, while only one instrument was specifically validated in patients with EGPA [26]. All instruments but one [20] were validated in an adult population. Overall, the instruments with strongest psychometric properties were the BVAS (all versions) for disease activity, the VDI for damage and the AAV-PRO for PRO/quality of life [20–22, 38, 40–42].

This systematic approach showed that the instruments developed or validated for vasculitis in general (such as the BVAS or VDI) or specifically for AAV (such as AAV-PRO), were those that performed the best [20–22, 38, 40–42], while the non-vasculitis or non-AAV-specific instruments performed on average worse, suggesting that active research in vasculitis is necessary to develop instruments optimally measuring disease domain(s) specific for AAV. The best example is for the AAV-PRO that, as compared with the other non-vasculitis specific HRQoL instruments, has high levels of almost all the properties assessed, i.e. the validity, reliability, responsiveness and feasibility, while the performance of SF-36, MFI-20 and PtGA ranged from low-to-moderate in the properties assessed [28, 43, 44]. However, AAV-PRO has not been used or validated in a clinical trial. In addition, these findings indirectly confirmed the expert-based opinion of the OMERACT group [4], which is reassuring.

### Assessment of disease activity

For disease activity, the BVAS/WG [41] performed better compared with BVAS.v3 [20], but the difference might lie in the fact that the first, specifically designed for GPA, was validated in patients with GPA, while the second was validated in all AAV. DEI showed adequate validity with good correlation with BVAS during active disease, and non-significant correlation with BVAS during disease remission [17]. The DEI aims to document organ involvement typically attributable to active vasculitis, which is linked to disease activity, while the BVAS measures disease activity, with the two measures providing complementary information. All these indices are highly correlated among themselves and in hands of experts, the instruments are highly reliable, as shown in an exercise comparing different AAV activity instruments [47]. ENT/GPA DAS, ENTAS and ENTAS 2, which assess organ-specific disease activity (i.e. the ENT domain) [11, 18, 19, 48], overall performed poorly. Interestingly, no EGPA-specific disease activity instruments were identified by this systematic review. Since psychometric features of BVAS were never validated in EGPA only, and it is believed that this tool does not adequately capture the full range of manifestations of EGPA, an EGPA-specific instrument would likely be a useful advance for the field.

Surprisingly, no studies have been found specifically assessing the role of PhGA in AAV, probably the most widely used measure in clinical practice. For some instruments, such as the BVAS, the PhGA is the major comparator to be correlated with the BVAS [41, 42]. In one study, PhGA among experts was collected in order to be compared with BVAS/WG, and thus inter- and intra-observer reliability determined (ICC of 0.96 and 0.28, respectively). These data are in contrast to other rheumatic diseases such as lupus, in which PhGA has been shown to be strongly influenced by the clinical experience of the physician, therefore producing a wide inter-observer variability, challenging comparison across patients [49]. In AAV, baseline PtGA–PhGA discordance was inversely associated with newly diagnosed disease (odds ratio 0.37, 95% CI 0.20, 0.68) [44]; however, no paper focussing on the psychometric properties of PhGA in AAV has been retrieved by this search.

### Assessment of disease damage

Not surprisingly, the VDI correlated weakly with disease activity and SF-36 [21]. As previously indicated by OMERACT, there are issues with content validity of the VDI as it may not detect all forms of damage incurred in AAV and items of damage not attributable to vasculitis are also recorded in the VDI, while surprisingly no reliability has been assessed. Therefore, research on other AAV-specific disease damage instruments is ongoing, as for ANCA-Vasculitis Index of Damage (AVID) (not yet validated) [50] and CDA [32], aiming to capture more items of damage than VDI.

### Assessment of HRQoL and other PRO

There has been growing interest in the importance of integrating patients’ perspectives on the impact of their disease, and quality of life has been proposed by OMERACT as a core domain to be assessed in clinical trials of AAV. Except for AAV-PRO, PRO or quality of life through different outcomes (fatigue, sleep, mental health, pain, physical and social functioning) was assessed using generic instruments not validated for AAV or for vasculitis in general. Among others, the SF-36 has been used; it covers eight domains, including physical and social functioning and mental health [51] and has been widely used in patients with inflammatory musculoskeletal disorders [52] and scantly in large vessel vasculitis [53]. In this review, SF-36 performed worse than AAV-PRO, and was often used as a comparator for other domains (such as VDI) [21]. VSMS, the other AAV-specific instrument to measure illness self-management, had a poor-to-moderate adequacy of validity [14].

### Assessment of function

Function was assessed with non-AAV-specific instruments, and all these instruments overall have a low-to-moderate performance. With the attempt to update and expand the OMERACT existing expert-driven core set for AAV [3, 5], several projects focussing on function in AAV have been evaluated [46]. As shown by this review, function remains an understudied domain in AAV, likely as a consequence of the numerous organs involved and the differences across the AAV subsets. Among others, ODSS [26], a validated score for immune-mediated polyneuropathies, has good inter-observer reliability, but only moderately adequate validity, even though it should be noted that ODSS has been retrospectively tested in a small population of EGPA patients (25 with peripheral neuropathy).

### Limitations and strengths

Surprisingly, the number of studies developed for or specifically assessing psychometric properties of the instrument assessing the OMERACT domains was relatively small. This might be a consequence of the eligibility criteria, which excluded studies that did not provide the performance of the psychometric measures of the instruments in AAV separately, in which a broader ‘vasculitis’ population including patients without AAV was studied, leading to the exclusion of some seminal papers, e.g. the ones describing the validation of the BVAS and VDI [54–57], other large validation studies [43, 58], or limiting the number of psychometric measures that can be extrapolated for AAV from the single studies [34, 38, 40–42]. Indeed, several studies, such as those assessing VAI, CDA, PROMIS, BVAS.v3, VDI, BIPQ and IPQ-R [14, 15, 29–32, 54–56], assessed numerous psychometric measures of these instruments tested in populations of patients with various forms of vasculitis and not only AAV, but rarely performed subset analyses on AAV only, therefore limiting the available data specific for AAV.

A strength of this study is that data were collected in a systematic literature review following state-of-the-art practices [8, 10]. A limitation is heterogeneity, since a single psychometric property of different instruments can be assessed in different ways, limiting the head-to-head comparability of their performances. Consequently, the evaluation of psychometric properties can be assessed with different methods (e.g. to evaluate reliability, Cohen’s K, ICC), with no direct comparability. A certain degree of heterogeneity is expected, since some instruments provide a single numeric score (e.g. BVAS, PtGA), others provide a profile (AAV-PRO, SF 36, MFI-20) and most are multi-item, although single-item instruments exist (PtGA).

### Conclusion

In conclusion, 22 instruments covering the OMERACT AAV core set domains of disease activity, damage, HRQoL/PRO and function had their psychometric properties assessed. Overall, the BVAS (any version), the VDI and the AAV-PRO were the instruments with the strongest psychometric properties. The majority of outcome instruments used for AAV were developed or validated for AAV as a group or GPA only, while specific studies for MPA or EGPA are lacking. The development and validation of outcome measurement instruments specific for AAV is warranted, since AAV-specific instruments are likely to capture a fuller range of disease manifestations yielding more precise measurements within the target disease, possibly assessing and reporting psychometric properties in a way that enables comparisons across instruments.

## Supplementary Material

keac175_Supplementary_Data

## Data Availability

The data underlying this article are available in the article and in its online supplementary material.
