# Supplementary material for: Psychometric properties of outcome measurement instruments for ANCA-associated vasculitis: a systematic literature review
Source: Rheumatology (Oxford). 2022 Mar 16;61(12):4603–18. doi: 10.1093/rheumatology/keac175 (PMC9707311; doi:10.1093/rheumatology/keac175)
Supplement: keac175_Supplementary_Data [file keac175_supplementary_data.docx]

**SUPPLEMETAL TABLES, FIGURES AND MATERIALS**

**SUPPLEMENTARY DATA S1: The PIM (Population, Instrument of interest, Measurement properties) question.**

| **POPULATION** | Patients with the following vasculitides: ANCA-associated vasculitis (granulomatosis with polyangiitis, eosinophilic granulomatosis with polyangiitis, microscopic polyangiitis, renal-limited vasculitis) |
| --- | --- |
| **INSTRUMENT OF INTEREST** | Disease Progression Index  Disease Exacerbation Index  Disease severity Index  Treatment Outcome Reproducibility of Results/Reliability/Validity  Physician Global Assessment (PhGA)  Patient Global Assessment (PGA)  Patient-reported outcomes (PRO) |
| **MEASURMENT PROPERTIES** | Psychometric properties (validity, reliability, feasibility, and responsiveness of the existing outcome measurements for ANCA-associated vasculitis |

**SUPPLEMENTARY DATA S2:** **Search Strategies**

**OVID**

Database(s): **Ovid MEDLINE(R) 1946 to Present and Epub Ahead of Print, In-Process & Other Non-Indexed Citations and Ovid MEDLINE(R) Daily, EBM Reviews - Cochrane Central Register of Controlled Trials** January 2020**, EBM Reviews - Cochrane Database of Systematic Reviews** 2005 to February 11, 2020**, Embase** 1974 to 2020 July 14

Search Strategy:

| 1 | exp Anti-Neutrophil Cytoplasmic Antibody-Associated Vasculitis/ |
| --- | --- |
| 2 | ((anca* or "anti-neutrophil cytoplasmic" or "antineutrophil cytoplasmic" or "renal limited" or "small vessel*" or systemic) adj3 (vasculit* or glomerulonephritis)).mp. |
| 3 | ((granulomatosis or microscopic) adj1 (wegener* or polyangitis)).ti. |
| 4 | "churg-Strauss syndrome".ti. |
| 5 | or/1-4 |
| 6 | Quality of Life/ or "quality of life".mp. |
| 7 | (disease adj3 (activity or active or extent or severity or assess*)).mp. |
| 8 | (organ* adj2 (involve* or damag*)).mp. |
| 9 | "patient reported outcome*".ti. |
| 10 | or/6-8 |
| 11 | (measur* or scor* or index* or survey* or instrument* or tool* or assess* or valid*).ti. |
| 12 | 10 and 11 |
| 13 | ("Short Form 36" or "short form-36" or "SF-36" or "Physician Global Assessment" or "Disease Extent Index" or "Five Factor* Score" or "systemic immune inflammation index" or "disease progression index" or "disease exacerbation index" or "disease severity index").mp. |
| 14 | Severity of Illness Index/ |
| 15 | Reproducibility of Results/ or reproducibility/ |
| 16 | Predictive Value of Tests/ or *predictive value/ |
| 17 | "Surveys and Questionnaires"/ |
| 18 | "Sensitivity and Specificity"/ |
| 19 | Health Status Indicators/ or health status indicator/ |
| 20 | Outcome Assessment, Health Care/ |
| 21 | Patient Reported Outcome Measures/ or patient-reported outcome/ |
| 22 | *Treatment Outcome/ |
| 23 | *outcome assessment/ |
| 24 | ("birmingham vasculitis activity score*" or "vasculitis damage index").mp. |
| 25 | or/13-24 |
| 26 | 12 or 25 |
| 27 | 5 and 26 |
| 28 | remove duplicates from 27 |

**SCOPUS**

| 1 | TITLE-ABS-KEY ((anca* or "anti-neutrophil cytoplasmic" or "antineutrophil cytoplasmic" or "renal limited" or "small vessel*" or systemic) w/3 (vasculit* or glomerulonephritis)) |
| --- | --- |
| 2 | TITLE ((granulomatosis or microscopic) w/1 (wegener* or polyangitis)) |
| 3 | TITLE ( "churg-Strauss syndrome" ) |
| 4 | 1 or 2 or 3 |
| 5 | TITLE-ABS-KEY ( "quality of life" ) |
| 6 | TITLE-ABS-KEY (disease w/3 (activity or active or extent or severity or assess*)) |
| 7 | TITLE-ABS-KEY (organ* w/2 (involve* or damag*)) |
| 8 | TITLE ( "patient reported outcome*" ) |
| 9 | 5 or 6 or 7 or 8 |
| 10 | TITLE (measur* or scor* or index* or survey* or instrument* or tool* or assess* or valid*) |
| 11 | 9 and 10 |
| 12 | TITLE-ABS-KEY ( "Short Form 36" OR "short form-36" OR "SF-36" OR "Physician Global Assessment" OR "Disease Extent Index" OR "Five Factor* Score" OR "systemic immune inflammation index" OR "disease progression index" OR "disease exacerbation index" OR "disease severity index" ) |
| 13 | TITLE (valid* or reproducib* or predict* or “treatment outcome*” or “outcome assessment” or “outcome measur*” or “severity of illness” or “health status indicator*” or (sensitivity w/1 specificity)) |
| 14 | TITLE-ABS-KEY ( "birmingham vasculitis activity score*" OR "vasculitis damage index") |
| 15 | 12 or 13 or 14 |
| 16 | 11 or 15 |
| 17 | 4 and 16 |
| 18 | INDEX(embase) OR INDEX(medline) OR PMID(0* OR 1* OR 2* OR 3* OR 4* OR 5* OR 6* OR 7* OR 8* OR 9*) |
| 19 | #17 and not #18 |
| 20 | DOCTYPE(ed) OR DOCTYPE(bk) OR DOCTYPE(er) OR DOCTYPE(no) OR DOCTYPE(sh) OR DOCTYPE(ch) |
| 21 | #19 and not #20 |

**SUPPLEMENTARY MATERIAL S3**: ***Search strategy and eligibility criteria***

Following the OMERACT Master Checklist for Instrument Selection filter 2.1 (Truth, Discrimination, Feasibility) and the COSMIN checklist frameworks, different psychometric properties were assessed: 1) validity (face validity, construct [group discrimination, hypothesis-testing or divergent/convergent validity], content and criterion validity), 2) reliability (internal consistency, inter- and intra-observer reliability), 3) responsiveness, 4) feasibility. Any type of development or validation studies dealing with psychometric properties of instruments used in AAV were assessed, including cross-sectional or longitudinal studies (for validity and reliability aspects), or randomized control trials (for responsiveness). If retrieved by the search, systematic reviews were used to identify the original studies.

**SUPPLEMENTARY MATERIAL S4: *Data extraction***

The following data were collected at the study level: year of publication, country, objective of the article (development/validation), type of study; specific quality aspects (blinding, number and time between observations, adequacy of the gold-standard, etc.); population subtype, sample size, and distribution of age and sex among patients.

The following data were collected at the instrument level: name and domains of the measurement tool, type (questionnaires, index, scale, biomarker, laboratory test, and imaging test), practical application (i.e., method of administration, score interpretation, cut-off points, smallest detectable change if described, completion time by the patient, scoring time by the assessor, strengths, and limitations), references, and psychometric properties (validity, reliability, feasibility, and responsiveness).

**Supplementary Figure S1. Flowchart of the systematic literature review.**

**
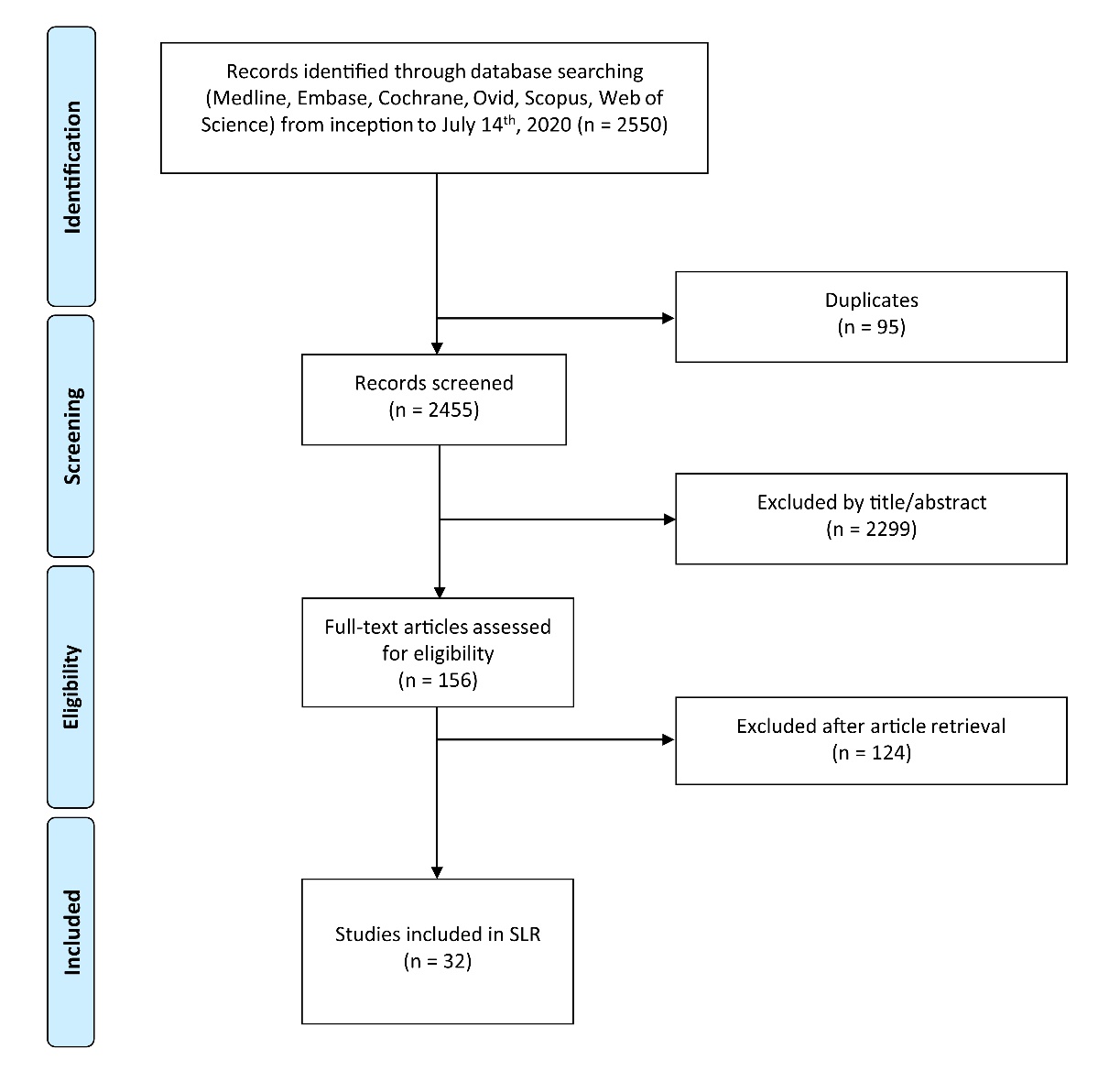
**

| **Supplementary Table S1.**  **Baseline characteristics of the populations of the 32 studies included.** | | | | | | | | |
| --- | --- | --- | --- | --- | --- | --- | --- | --- |
| **Author** | | **Year** | **Instrument** | **Population*** | **Sample size (# subjects with AAV)** | **Age** | **Female (%)** | **Country** |
| Annapureddy N. | | 2016 | **RAPID3** | GPA, MPA, EGPA | 34 | 55.15 (26-84) | 82.4% | USA |
| de Groot, K. | | 2001 | **DEI** | GPA | 66 | NR | 51.5% | Germany |
| Garske U. | | 2012 | **ENTAS** | GPA | 45 | 56±17 | 51.1% | Germany |
| Decker L. | | 2017 | **ENTAS2** | GPA | 47 | 56±15 | 63.8% | Germany |
| Del Pero M. | | 2013 | **ENT/GPA DAS** | GPA | 144 | 57.7 (42.5-68.5) | 47.9% | UK |
| Mahr, A. | | 2008 | **BVAS/WG** | GPA | 180 | 47±16 | 40.0% | USA |
| Stone J. | | 2001 | **BVAS/WG** | GPA | 117 | NR | NR | USA |
| Morishita K. | | 2012 | **BVAS.v3** | GPA, MPA, EGPA, unclassified* | 152 | NR | 68.4% | USA, Canada |
| Seo P. | | 2005 | **VDI** | GPA | 180 | 47±16 | 40.0% | USA |
| Itabashi M. | | 2014 | **VDI** | MPA | 30 | 62±14 | 63.3% | Japan |
| Kim M.K. | | 2020 | **MVIA** | GPA, MPA, EGPA | 182 | 58.5±21.0 | 69.8% | Japan |
| Robson JC. | | 2018 | **AAV-PRO** | GPA, MPA, EGPA | 50 | 80%≥50 years old | 52.0% | USA, UK Canada |
| Robson JC. | | 2018 | **AAV-PRO** | GPA, MPA, EGPA | 626 | 60.4±13.2 | 64.0% | USA, UK, Canada |
| Tomasson G. | | 2012 | **SF-36** | GPA | Cohort 1: 180  Cohort 2: 228 | Cohort 1: 49.9±15.4) Cohort 2: 49.1±16.7 | Cohort 1: 40.0% Cohort 2: 52.6% | USA, Europe |
| Tomasson G. | | 2014 | **PtGA** | GPA | 180 | 47±16 | 40.0% | USA |
| Moog P. | | 2016 | **COMPASS31** | GPA, MPA | 27 | 53 (21–80) | 48.1% | Germany |
| Koutantji M. | | 2003 | **HAQ** | GPA, MPA, EGPA | 51 | 63.06±10.13 | 41.0% | UK |
| Padoan R. | | 2018 | **ODSS** | EGPA^1^ | 50 | 49.98±13.86 | 38.0% | Italy |
| Faurschou M. | | 2010 | **SF-36** | GPA | 68 | 58 (17-78) | 47.0% | Denmark |
| McClean A. | | 2016 | **MFI-20** | GPA, MPA | 48 | 58 (47–65) | 46.0% | UK |
| Thorpe C. T. | | 2007 | **VSMS** | GPA, EGPA, MPA^2^ | 205 | 54.7 ± 14.7 | 53.7% | USA |
| Schwartz M.N. | | 2020 | **BIPQ** | GPA, EGPA, MPA^3^ | 46 | 53±18 | 56.5% | USA |
| Grayson P.C. | | 2014 | **IPQ-R,**  **MFI-20** | GPA, EGPA, MPA | 495 | EGPA: 55.0 (25–80) GPA: 54.8 (26–86) MPA: 57.7 (25–76) | EGPA: 69% GPA: 63% MPA: 82% | USA |
| Whiting-O'Keefe Q. E. | | 1999 | **VAI** | GPA, EGPA, MPA^4^ | 41 | NR | NR | USA |
| Suppiah R. | 2011 | | **BVAS.v3** | GPA, MPA, EGPA^5^ | 194 | GPA (renal): 56 (17-85) GPA (non-renal): 53 (19-75) EGPA:68 (45-82) MPA: 56 (17-81) | GPA (renal): 40% GPA (non-renal): 51% EGPA:52% MPA: 45% | Europe |
| Suppiah R. | 2011 | | **CDA** | GPA, EGPA, MPA^6^ | 220 | NR | NR | Europe |
| Robson J. | 2015 | | **VDI** | GPA, MPA | 735 | 57.6 (14.4) | 43.50% | Europe |
| Specks U. | 2013 | | **BVAS,**  **VDI, SF-36** | GPA, MPA | 197 | RTX: 54.0±16.8  CYC: 51.5±14.1 | RTX: 54%  CYC: 46% | USA |
| Yumura W. | 2014 | | **BVAS** | MPA | 44 | By severity:  most severe (n=2): 66.5 (62-71) severe (n=23): 67.6 (56-76) mild (n=23): 65.7 (26-79) | By severity:  most severe (n=2): 100% severe (n=23): 48% mild (n=23): 78% | Japan |
| Metzler C. | 2007 | | **BVAS,**  **DEI** | GPA | 48 | LEF: 55(27-56)  MTX: 54 (25-67) | LEF: 38%  MTX: 43% | Germany |
| Monach P. | 2011 | | **BVAS** | GPA, MPA | 146 | RTX: 54.0±16.8  CYC: 51.5±14.1 | RTX: 54%  CYC: 46% | USA |
| Tomasson G. | 2019 | | **PROMIS** | GPA, EGPA, MPA^7^ | 629 | 57.1±16.3 | 62.1% | USA |

*Abbreviation: AAV=ANCA-Associated Vasculitis; GPA=Granulomatosis with Polyangiitis; MPA= Microscopic Polyangiitis; EGPA= Eosinophilic Granulomatosis with Polyangiitis; RTX=Rituximab; CYC=Cyclophosphamide; MTX=Methotrexate; LEF=Leflunomide; NR=non reported.QoL/PRO: quality of life/patient reported outcomes. Birmingham Vasculitis Activity Score (BVAS) version 3 (BVAS.v3) and BVAS for Wegener Granulomatosis (BVAS/WG), Ear nose and throat (ENT)/GPA disease activity score (ENT/GPA DAS), Disease Extent Index (DEI), multivariable index for AAV (MVIA) and Vasculitis activity index (VAI); vasculitis damage index (VDI), Combined Damage Assessment Index (CDA), ENT assessment score (ENTAS) and its newer version ENTAS 2, AAV-patient reported outcome (AAV-PRO), Vasculitis Self-Management Scale (VSMS), Study Short-Form 36 (SF-36), Multidimensional Fatigue Inventory-20 (MFI-20), Patients global assessment (PtGA), Brief Illness Perception Questionnaire (BIPQ), revised Illness Perception Questionnaire (IPQ-R), Routine assessment of patient index data 3 (RAPID3), Health Assessment Questionnaire (HAQ), Overall Disability Sum Score (ODSS) and Composite Autonomic Symptom Score 31 (COMPASS31).*

* In all cases but one, the study population is adult. Morishita K. is conducted on a pediatric population

^1^ 25 patients with neurological involvement

^2^ 1 patient Goodpasture and 2 GCA patient included in the cohort (total 205 patients)

^3^ 196 patients: Giant cell arteritis=47, Takayasu arteritis=47, Relapsing polychondritis=56, AAV=46

^4^ 74 patients: Relapsing polychondritis=4, Polyarteritis nodosa=13, MPA=3, EGPA=3, GPA=35, Behçet's Disease=7, Cryglobulinemic vasculitis=2, other systemic necrotizing vasculitis=7

^5^ 238 patients: GPA=98 (renal), GPA (non renal)=51, EGPA=23, MPA=22, other=13, Henoch-Schönlein purpura=7, mixed essential cryoglobulinemia=9, Takayasu arteritis =6, Behçet's Disease=5, leucocytoclastic skin vasculitis=2, Polyarteritis nodosa=2.

^6^ 283 patients: GPA (renal)=104, GPA (non-renal)=61, MPA=31, EGPA=24, Henoch-Schonlein purpura=11, Mixed cryoglobulinemia11, Behçet's Disease=9, Takayasu arteritis =7, Isolated skin vasculitis=4, Polyarteritis nodosa=2, systemic rheumatoid vasculitis=2, Other=24.

^7^ 973 patients: GPA=433, EGPA=123, MPA=73, Giant cell arteritis=194, Takayasu arteritis=97, Polyarteritis nodosa=53.
